# Altered levels of circulating nuclear and mitochondrial DNA in patients with Papillary Thyroid Cancer

**DOI:** 10.1038/s41598-019-51000-7

**Published:** 2019-10-08

**Authors:** Ewelina Perdas, Robert Stawski, Krzysztof Kaczka, Dariusz Nowak, Maria Zubrzycka

**Affiliations:** 1 Department of Cardiovascular Physiology, Faculty of Medicine, Medical University of Lodz, Lodz, Poland; 2 Department of Clinical Physiology, Faculty of Medicine, Medical University of Lodz, Lodz, Poland; 3 Department of General and Oncological Surgery, Medical University of Lodz, Lodz, Poland

**Keywords:** Thyroid cancer, Thyroid cancer

## Abstract

Papillary thyroid cancer is the most common thyroid cancer type. However, diagnostics based on fine needle biopsy cannot make a definitive diagnosis in 25% of thyroid nodules. Additionally, approximately 70% to 80% of thyroid lobectomies performed just for diagnostic purposes are benign. Despite this, biopsy still remains the main method of evaluation of thyroid nodules. Cell-free DNA (cf-DNA) measurement could give a new diagnostic opportunities which may reduce the number of unnecessary thyroid procedures. In this study, using a qPCR, we have examined the nuclear cf-DNA and mitochondrial cf-DNA in the plasma of 32 patients. We have found that the level of nuclear cf-DNA is almost 2-fold increased (median 3 089 vs. 1 872, p = 0.022), whereas mitochondrial cf-DNA content was significantly decreased in respect to healthy controls (median 44 992 vs. 92 220, p = 0.010). The ROC curve analysis showed high specificity for nuclear cf-DNA and mitochondrial cf-DNA, which may serve as a useful tool to decrease the number of unneeded surgeries. Our study reports the first epidemiological evidence for lower mitochondrial cf-DNA content in the patient group, what suggests that apart from nuclear cf-DNA also mitochondrial cf-DNA is affected by disease development.

## Introduction

Papillary thyroid cancer (PTC) is the most common thyroid cancer type accounting for 80% of all cancers of the thyroid gland. The incidence of PTC has increased 3-fold over the last 30 years^1^. Thyroid cancer is associated with 5-year disease-specific survival rate of 98%, with a nearly 100% survival rate for the 68% of patients diagnosed with localized disease^2^.

Current established methods for routine thyroid cancer screening first include non-invasive methods that include imaging techniques such as ultrasonography. However, to achieve an ultimate diagnosis before deciding on treatment, these techniques generally have to be followed by histopathological analysis for which invasive procedures, such as a fine needle biopsy (FNB) are necessary^3^. However, FNB cannot make a definitive diagnosis in 25% of thyroid nodules. Approximately 70% to 80% of thyroid lobectomies performed just for diagnostic purposes are benign^4^. Furthermore, FNB still remains the main method of evaluation of thyroid nodules.

Molecular testing may reduce the number of unnecessary thyroid procedures. Lately, the clinical view on total circulating DNA has sparked the interest of scientists as it opens up a new possibility for non-invasive monitoring. A diagnostic opportunity is the measurement of total circulating cell-free DNA (cf-DNA) levels in cancer.

The circulating DNA is composed of nuclear DNA (cf-nDNA) and mitochondrial DNA (cf-mtDNA) deriving from nucleus or cytoplasmic mitochondria, respectively. Mitochondria carry their own extrachromosomal genome, which exists at hundreds to thousands of copies within a single mammalian cell as opposed to two copies of genomic DNA and thus mtDNA levels exceed those of nDNA several-fold. Mitochondrial DNA replicates independently of nuclear nDNA^5^. Both nDNA and mtDNA have become a matter of investigation and qualitative as well as quantitative alterations in these two determinants have been implicated in cancer^6^. Changes in the level of cf-nDNA and cf-mtDNA have been found in plasma and serum of patients with various cancer types^7^.

Both of these forms are released by malignant tissues and also by surrounding tissues suffering from malnutrition, hypoxia, or other factors associated with cancer development. Moreover, cancer patients generally have much higher levels of cf-nDNA than healthy individuals, but the levels vary widely. The variability of cf-nDNA levels in cancer patients is likely to be associated with the tumor burden, stage, cellular turnover, and response to therapy. In contrast to cf-nDNA, cf-mtDNA increase is not so common, it also frequently remains unchanged or decreases.

In this study, using a real-time PCR, we have examined the cf-nDNA and cf-mtDNA in the plasma of patients with PTC, and compared them with those of a healthy control group to investigate whether cf-nDNA and cf-mtDNA also have a value in the management of PTC.

## Results

### Comparison of plasma cf-nDNA and cf-mtDNA levels between the study groups

We compared the levels of cf-nDNA and cf-mtDNA in plasma, by real-time PCR, between the PTC cases and healthy subjects. The median age for cancer patients (n = 32) was 49 years and the median age of healthy control individuals (n = 30) was 47 years.

The overall level of cf-nDNA in the PTC group was significantly higher in comparison with the healthy control group (median 3 089 vs. 1 872, Mann-Whitney: p = 0.022). The level of cf-nDNA in the PTC subgroup at, or above, the median age was significantly higher in comparison with healthy control subgroup at, or above, the median age (median 3 421 vs. 1 981, Mann-Whitney: p = 0.018). There were no statistically significant differences in the level of cf-nDNA in the PTC subgroup below the median age in comparison with healthy control subgroup below the median age (median 2 631 vs 1 568, Mann-Whitney: p = 0.308). Similarly, for cf-nDNA level no statistical change was found in the control group itself as well as in the PTC group.

The level of cf-nDNA among males in the PTC group was significantly higher in comparison with males from the healthy control group (median 3 900 vs. 1 364, Mann-Whitney: p = 0.023). There were no statistically significant differences in the level of cf-nDNA among females in the PTC group in comparison with females of the healthy control group (median 2 700 vs. 2 307, Mann-Whitney: p = 0.153). Similarly, for cf-nDNA level no statistical difference was found between males and females in the PTC group and the control group (median 3 900 vs 2 700, Mann-Whitney: p = 0.186 and median 1 364 vs 1 364, Mann-Whitney: p = 0.414, respectively). The data are presented in Table 1.

**Table 1:** Levels of cf-nDNA in study groups.

Group	Control (GE/ml) n = 30 (M = 11 F = 19)	PTC (GE/ml) n = 32 (M = 5 F = 27)
Overall	1 901 ± 931 (1 872)	5 235 ± 10 934 (3 089)*
**Age**		
<median	1 728 ± 916 (1 568)	2 587 ± 1 881 9 (2 375)
≥median	2 052 ± 947 (1 981)	3 906 ± 3 390 (3 421)*
**Gender**		
Male	1 734 ± 1 052 (1 364)	15 409 ± 26 662 (3 900)*
Female	1 997 ± 869 (2 307)	3 350 ± 3 095 (2 700)

Data are expressed as mean ± standard deviation (median). GE-genome equivalents. ^*^Statistically significant data corresponding control, p ≤ 0.05.

The overall level of cf-mtDNA in the PTC group was significantly lower in comparison to the healthy group (median 44 992 vs. 92 220, Mann-Whitney: p = 0.010). The level of cf-mtDNA in healthy control subgroup below the median age were no statistically significant differences in comparison with the PTC subgroup below the median age (median 104 129 vs. 41 161, Mann-Whitney: p = 0.013). There were no statistically significant differences in the level of cf-mtDNA in in the PTC subgroup at, or above, the median age in comparison with healthy control subgroup at, or above, the median age (median 57 899 vs. 92 172, Mann-Whitney: p = 0.418). Similarly, for cf-mtDNA level no statistical change was found in the control group itself as well as in the PTC group.

The level of cf-mtDNA among females from the healthy control group was significantly higher in comparison with females from the PTC group (median 92 220 vs. 43 067, Mann-Whitney: p = 0.008). A statistical difference was found between males and females in the PTC group (192 956 vs. 43 067, Mann-Whitney: p = 0.038). There were no statistically significant differences in the levels of cf-mtDNA among males in the PTC group in comparison with males from the healthy control group (median 192 956 vs 92 124, Mann-Whitney: p = 0.821). Similarly, for cf-mtDNA level no statistical difference was found between males and females in the control group (median 92 124 vs 92 220, Mann-Whitney: p = 0.576). The data are presented in Table 2.

**Table 2:** Levels of cf-mtDNA in study groups.

Group	Control(GE/ml) n = 30 (M = 11 F = 19)	PTC(GE/ml) n = 32 (M = 5 F = 27)
Overall	176 760 ± 255 401 (92 220)	110 664 ± 186 943 (44 992)^*^
**Age**		
<median	162 525 ± 159 078 (104 129)	135 446 ± 247 577 (41 161)
≥median	189 216 ± 322 218 (92 172)	85 882 ± 98 086 (46 868)
**Gender**		
Male	184 185 ± 173 237 (92 124)	341 541 ± 390 531 (192 956)^#^
Female	172 462 ± 297 265 (92 220)	67 909 ± 77 817 (43 067)^*^

Data are expressed as mean ± standard deviation (median). GE-genome equivalents. ^*^Statistically significant data corresponding control p ≤ 0.05. ^#^Statistically significant data corresponding female subgroup, p ≤ 0.05.

### Tumor and lymph nodes characteristics in PTC group

Classification of the primary tumor (T) and regional lymph nodes (N) was carried out according to the system accepted by the International Union Against Cancer (UICC, 2010).

The level of cf-nDNA as well as cf-mtDNA was higher in the case of less advanced tumors, the difference is statistically insignificant, median 3 331 vs. 2 700 Mann-Whitney: p = 0.675 and median 46 598 vs. 38 937 p = 0.801, respectively.

The median level of cf-nDNA, as well as the level of cf-mtDNA, was lower in the group of patients with regional lymph node metastases (N_1_) compared to no regional lymph nodes metastases (N_0_). Median 3 511 vs. 2 700, Mann-Whitney: p = 0.633 and 47 138 vs. 43 386, Mann-Whitney: p = 0.766, respectively. In the group where lymph nodes could not be assessed (N_x_) median level of cf-nDNA was comparable to the N_1_ group (median 3 036 vs. 3 511) but higher than in N_0_ (median 2 700). The difference is statistically insignificant between these three groups (Kruskal-Wallis: p = 0.831). The median level of cf-mtDNA was lowest in the N_x_ group compared to the N_0_ and N_1_ groups (37 790 vs. 47 138 vs. 43 386 Kruskal-Wallis: p = 0.662). The data are presented in Table 3.

**Table 3:** Comparison of cf-nDNA & cf-mtDNA and the primary tumor (T) and regional lymph nodes (N).

T&N staging	n	cf-nDNA (GE/ml)	cf-mtDNA(GE/ml)
T_1_ + T_2_	23	5 820 ± 12 665 (3 331)	127 557 ± 216 542 (46 598)
T_3_ + T_4_	9	3 738 ± 4 318 (2 700)	67 492 ± 59 635 (38 937)
N_0_	15	3 250 ± 2 462 (3 511)	84 952 ± 94 747 (47 138)
N_1_	9	3 776 ± 4 194 (2 700)	114 292 ± 141 680 (43 386)
N_X_	8	10 596 ± 21 288 (3 036)	154 793 ± 331 986 (37 790)

Data are expressed as mean ± standard deviation (median). GE-genome equivalents.

### Receiver operating characteristic (ROC) curve

**Figure 1:**
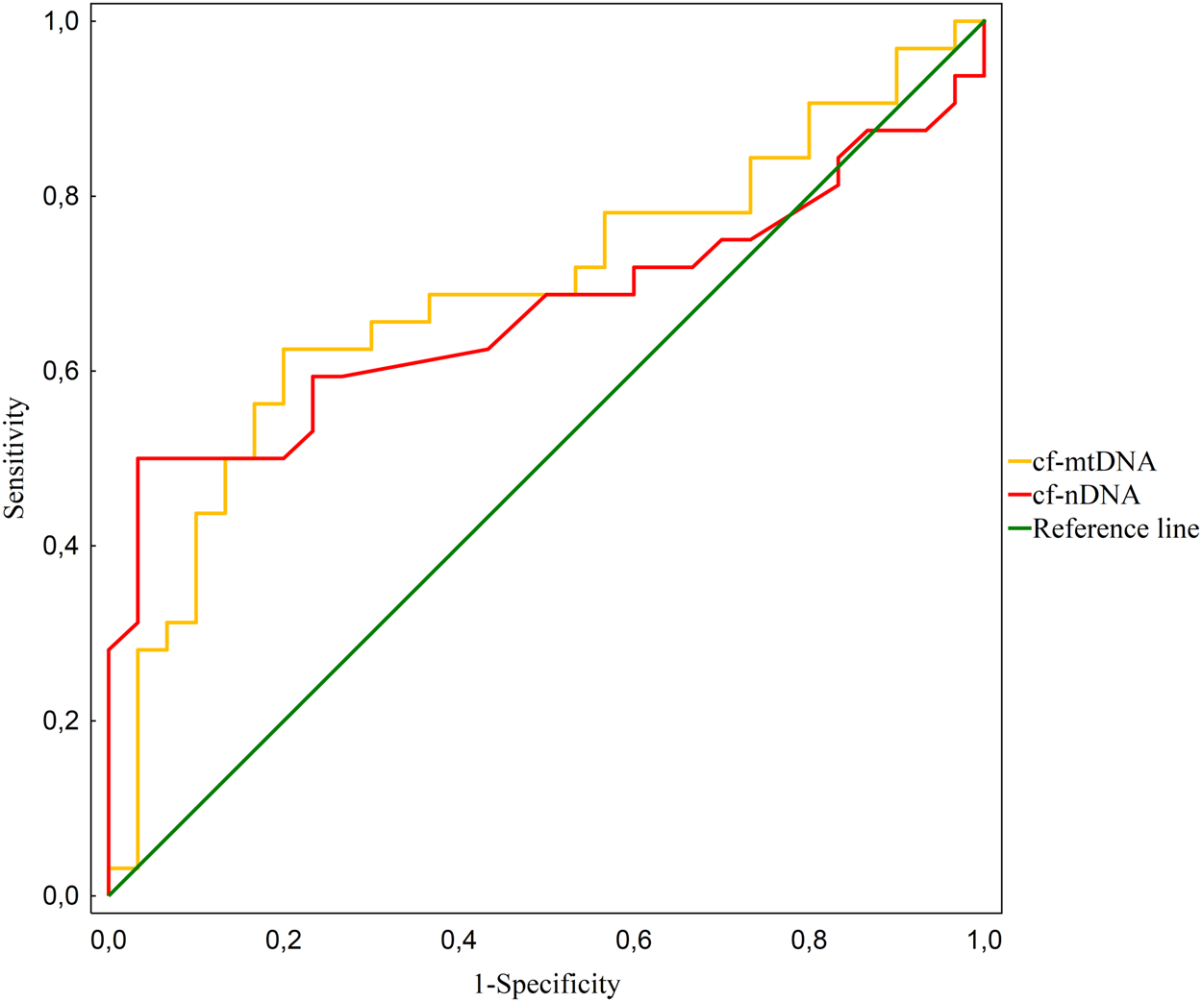
Receiver-operator characteristic (ROC) curves considering all the samples from the training data set (32 cancer cases and 30 controls) for the cf-nDNA and cf-mtDNA. The ROC curves plot sensitivity versus 1- specificity. The determined cut-off values for cf-nDNA and cf-mtDNA were 3 331 and 56 292, respectively.

Receiver operating characteristic (ROC) curve analyses were performed to evaluate the diagnostic utility of cf-nDNA and cf-mtDNA in the PTC versus healthy groups. The areas under the ROC curves (AUCs) were measured to calculate the specificity and sensitivity of cf-nDNA and cf-mtDNA to diagnose patients with PTC. The Youden index (J) allows the selection of an optimal cut-off point for discriminating between the PTC group and the healthy control group. J is the maximum vertical distance between the ROC-curve and the diagonal reference line and is defined as J = maximum (sensitivity) + (specificity) − 1. The data are illustrated in Fig. 1.

The level of cf-nDNA in the PTC cases was significantly higher in comparison with the healthy controls. An optimal cut-off point was indicated at 3 331 GE/ml for plasma cf-nDNA with a sensitivity of 50% and a specificity of 96% (AUC = 0.67, p < 0.05, 95% confidence interval = 0.527–0.808).

An decreased level of cf-mtDNA was observed in the PTC group when compared to the controls. For plasma cf-mtDNA an optimal cut-off point was indicated at 56 292 GE/ml with a sensitivity of 62% and a specificity of 80% (AUC = 0.69 p < 0.05, 95% confidence interval = 0.556–0.825).

## Discussion

In the present study, we have evaluated the changes of cf-nDNA and cf-mtDNA in plasma samples from a cohort of PCT patients compared to healthy controls. The cf-nDNA is present in the plasma and serum of healthy subjects, however, its level is usually much higher in people after physical effort, patients with various benign diseases such as trauma, stroke, burns, sepsis, autoimmune diseases, and also in patients with some cancers^8,9^. The mechanism of cf-nDNA release is unclear. Biological processes involved in cf-nDNA release include lysis, apoptosis, necrosis, and/or spontaneous active release from circulating tumor cells. In general, cancer pathogenesis is associated with increased apoptosis and necrosis of the tumor itself or the surrounding tissues^10^, or non-malignant host cells from the immune system, all of the above might produce cf-nDNA in response to malignant environment^11^. Moreover, certain cancers can resist apoptosis or necrosis until they undergo treatment and chemotherapy.

In our study, we have noticed a significant increase of cf-nDNA. The observed change is in the line with other PTC studies of Zane, *et al*.^12^, an increase from 5.14 ng to 22 ng and a 2-fold change reported by Salvianti, *et al*.^13^. A two-fold change has been also observed in ovarian cancer^14^. In breast cancer, the observed increase was 1298 GE/ml in control vs. 4678GE/ml in malignant tissue. In colorectal cancer, the increase ranged from 2- to 10-fold^15^. Kumar, *et al*.^16^ has observed an increase from 1650 GE/ml to 5451 GE/ml in head and neck squamous-cell carcinoma (HNSCC) patients. Hence, despite the moderate malignancy of the PTCs, the cell free growth is comparable with other types of cancer.

The cf-mtDNA is a much less common target for scientific efforts compared to cf-nDNA. Furthermore, cf-mtDNA is not always correlated with cf-nDNA levels, and in certain pathological conditions, providing potentially unique information distinct from that from cf-nDNA. For instance, a significant difference between the epithelial ovarian cancer and endometriosis groups was found only in cf-mtDNA but not in cf-nDNA^14^. While the rise of cf-nDNA suggests the disease progression or cellular stress, the causes of changes in cf-mtDNA level are not so obvious. The release of cf-mtDNA into circulation might be due to an immunological process but also might be related to cellular metabolism or maintenance. Recent paper have shown that cf-DNA might be produced in neutrophil extracellular traps (NETs). During NETs formation, the neutrophil releases its DNA which mainly consists from nucleic acid originated from mitochondria towards the pathogen^17^. Moreover, a plasma cf-mtDNA increase has been observed in endometriosis or lung cancer and it is usually explained by compensatory mechanism of the cells in response to the decline in respiratory function or redox imbalance^18,19^.

In other cancer studies, similarly to our results, cf-mtDNA was more commonly found to decrease in plasma. The decline observed in our PTC patients from 176 760 GE/ml to 110 664 GE/ml was consistent with the decrease observed in breast cancer - from 522 115GE/ml to 205 013 GE/ml^7^. Furthermore, in HNSCC the decline was from 29 100 000 vs. 9 200 000 GE/ml^16^. To our knowledge, there are currently no studies that have examined cf-mtDNA content in PTC. Noteworthy, the decrease in cf-mtDNA is inconsistent with microscopic observation of the thyroid cancer, where the cancerous tissue contains increased number of mitochondria^20^. Moreover, Su, *et al*.^21^ using RT-PCR showed that the average content of mtDNA copies number in PTC tumors was nearly four times higher than that in the adjacent normal tissues. However, a similar phenomenon has been observed in breast cancer, where the increase in tissue mtDNA copies number conflicts with a decrease of cf-mtDNA^22^. There is also evidence that cancer tissue mitochondria might be morphologically changed^23^. In this context, the question remains, if they are functioning properly to protect their DNA integrity and release.

The decrease of cf-mtDNA is explained by mutations in mitochondrial DNA, which are characteristic of the pathogenesis of various tumors. Those mutations might be caused by the fact that unlike nuclear DNAs, mtDNA does not have protective histones and sophisticated DNA repair mechanisms, which makes them extremely susceptible to oxidative stress. The mitochondrial DNA is exposed to reactive oxygen species (ROS) which are a by-product of respiration occurring in mitochondria. This environment of high oxidative stress contributes to the high predisposition of mtDNA to mutagenesis. It was proposed that point mutations adjacent to the replication region in D-loop the non-coding regulating both replication and transcription of the mitochondrial genome, may play a role in reduction of mtDNA in cancer^24^. In thyroid cancer, mtDNA D-Loop somatic mutations were detected in almost fifty percent of the tumors, both benign and malignant, thus supporting the assumption that these alterations are early and common events in thyroid carcinogenesis^25,26^. Moreover, the estrogen receptor α (ERα) has been identified in mitochondria and to bind to the D-loop of mtDNA^27^. This may suggest that ERs mediate mtDNA transcription. Research findings show that low mtDNA level resulted in increased ERα expression in PTC compared to control^28^. Changes in ER protein expression have been described to be involved in PTC tumor biology. Fan, *et al*.^29^ and Vannucchi, *et al*.^30^ also have reported that the expression of ERα protein was higher in PTC compared with matched non-PTC thyroid controls from same patients. These findings indicate that ERα overexpression may stimulate the development of PTC. It is worth noting that the works focus on women only^28^, or the work does not take into account the division into sex groups^30^, or the male group is small in relation to women (8/41)^29^. It is not clear whether ERα directly regulates mtDNA transcription, or whether this effect is mediated through the nuclear effects on nuclear-encoded genes that in turn regulate mtDNA transcription and whether it is gender-related. An alternative explanation for the decreased mtDNA content in tumors tissues with increased tumors size could be that the ineffective mtDNA biosynthesis is unable to make up with the enhanced cellular proliferation in tumors^31^.

Interestingly, cf-mtDNAs have presented a gender bias. In our research, the cf-mtDNA level was significantly reduced in the group of women compared to controls and between men and women in the PTC group. Moreover, where cf-mtDNA decreases in general, in male patients we observed a high, however statistically insignificant increase. It is very difficult to draw conclusions from these results, because of a very small cohort of PTC male patients. Despite this, the results are very promising and the research will be continued in a larger patient group. This supports our hypothesis that thyroid cancer, or maybe also other gender-specific malignancies should be considered in a gender-specific manner^32,33^. The gender disparities in thyroid cancer incidence, age of occurrence and prognosis are well established, however, the understanding of the molecular factors that mediate this difference is still poor. A detailed understanding of the effects of ER expression and its effects on mtDNA in PTC is also required to study potential targeted therapies that may modulate ER actions in PTC pathogenesis. Whether ER expression changes occurring in PTC in men and its consequences for PTC pathogenesis also requires further investigation. Such information may lead to new therapeutic approaches. The noted difference in cf-mtDNA in male patients was statistically significant despite the limited number of cases. These preliminary results are very promising and should be elucidated in other studies and larger patient cohorts.

Noteworthy, in our study the overall growth of cf-nDNA was mainly generated by male PTC cases which accounted for almost 3-fold increase. This should be considered in the context that thyroid cancer predominantly affects women, although it carries worse prognosis and higher mortality in men^33^. However, none of the previous PTC studies have observed such correlation^12,13^.

The ROC curve is a fundamental tool for diagnostic test evaluation. It is a plot of the true positive rate (Sensitivity) against the false positive rate (1-Specificity) for different cut-off points of a parameter. In our study, after ROC analysis we found that specificity was 96% and 80%, whereas sensitivity was 50% and 62%, respectively for cf-nDNA and mt-DNA. For some reasons, quantitative and qualitative cf-nDNA and cf-mtDNA provide satisfying specificity, though sensitivity seems to be relatively low to discriminate consistently between oncogenic and healthy individuals. Moreover, combined analyses of cf-nDNA and cf-mtDNA did not improve the specificity or sensitivity parameter (data not shown). This might be due to variability of the cf level in healthy controls (Table 2) which is present in the circulation of healthy individuals and the physiological factors which influence the levels of cf-DNA are unclear, which makes it difficult to establish a clear baseline and to interpret the results correctly. To sum up, the relatively high specificity still allows to decrease the number of unneeded surgeries.

While the cf-nDNA results were quite prospective and in the line with other studies, ones concerning cf-mtDNA are newer and very promising. This study is the first to report epidemiological evidence for lower cf-mtDNA content in cell free plasma samples in PTC patients. Furthermore, we first reported the gender bias where male patients have higher cf-nDNA compared to females. It might suggest that cf-DNA is a very sensitive marker of even small changes in tumor aggressiveness. However, no correlations with the tumor grade or stage have been observed. Also in accordance with other publications, no associations with histopathological diagnosis or age have been observed. In the context of limited number of tumors originating form male patients and the reported gender bias in both forms of cf-DNA, we advise caution in interpreting these results and consider them rather as preliminary results.

### Final remarks

In conclusion, our study reports the first epidemiological evidence for lower cf-mtDNA content in the PTC patient group. Furthermore, our results were confirmatory in observation of the increase of cf-nDNA in PTC patients plasma samples. The finding that the level of cf-nDNA is significantly increased, whereas cf-mtDNA content was significantly decreased, is consistent with the reports concerning other tumors. In this respect, we assume that both forms of cf-DNA should be considered as an option in PTC diagnostics as a prognostic factor or/and a biomarker. However, our observation requires further research.

## Methods

### Studied population

The presence of cf-nDNA and cf-mtDNA in plasma was evaluated in patients admitted to the Department of General and Oncological Surgery of the Medical University of Lodz. The patients were suspected of, or diagnosed with, papillary thyroid cancer based on fine needle aspiration biopsy (FNAB) before the surgery. The surgical procedure was performed on all patients in accordance with the guidelines of the Polish Society of Surgeons and the Polish Society of Oncological Surgery^3^. A total of 32 patients (27 females and 5 males) was enrolled. The inclusion criteria were: (1) adult patient of both sexes in age between 20 and 80 years, (2) papillary thyroid cancer confirmed histologically, (3) a written informed consent before initiating study procedure. The exclusion criteria were: (1) alcohol and illicit drug abuse, (2) history of infectious and inflammatory disease within 3 months prior to the study, (3) history of other malignancy, (4) pregnancy, (5) treatment with anti-inflammatory, immunosuppressive drugs within 3 months prior to the study. Staging of the tumors were was out according to the system approved by the International Union Against Cancer (UICC, 2010) and/or WHO classification. The 30 control subjects (19 females and 11 males) were healthy volunteers. For details see Supplementary Tables S1 and S2. The protocol was reviewed and approved by The Medical University of Lodz Ethics Committee (RNN/146/18/KE dated May 15th, 2018) and in compliance with the Declaration of Helsinki. All patients and volunteers provided written informed consent.

### Blood sample processing and cf-DNA extraction

From each patient, 9 mL venous blood was collected in vacutainer tubes (Becton Dickinson, Franklin Lakes, NJ) with EDTA for DNA extraction and analysis for cf-nDNA and cf-mtDNA using real time PCR analysis. The samples were collected before surgery and processed within 2 h after venipuncture.

Plasma from EDTA blood samples was obtained by centrifugation (1600 x g, 4 °C) for 10 min. Then, plasma samples were subjected to the second centrifugation (16 000 × g, 4 °C, 5 min) to remove the cell debris and were stored at −80 °C for no longer than 4 weeks until cf-DNA level measurements were obtained. Cell free DNA (cf-nDNA and cf-mtDNA) was isolated from 200 μL plasma using a QIAamp DNA Blood Mini Kit (Qiagen GmbH, Hilden, Germany) according to the manufacturer’s instructions with elution into 50 μL TE buffer. Purity of all samples was examined using Picodrop Spectrophotometer and 260/280 ratio was in the acceptable range 1,7–2,0.

### Real-time PCR for the measurement of cf-nDNA and cf- mtDNA in plasma

For the quantification of cf-nDNA and cf-mtDNA isolated from plasma, a quantitative real-time PCR (qPCR) based on TaqMan gene expression assay was performed, in the “CoreLab” Central Scientific Laboratory of the Medical University of Lodz, using the glyceraldehyde 3-phosphate-dehydrogenase (GAPDH) gene for cf-nDNA and mitochondrially encoded ATP synthase 8 (MT-ATP 8) gene for the cf-mtDNA according to the method described previously^7,9^ (see Supplementary Table S3). Negative control samples received 2 μL of TE buffer without cf-DNA from plasma. The reaction was performed in duplicate using the 7900 HT Real-time PCR System (Applied Biosystems) under the following conditions: an initiation step at 50 °C for 2 min, followed by the first denaturation at 95 °C for 10 min, then 40 cycles of 95 °C for 15 s and annealing at 60 °C for 1 min.

Individual results were obtained as a mean from two separate runs and expressed as genome equivalents (GE)/ml plasma (1 GE = 6.6 pg DNA) for cf-nDNA and cf-mtDNA.

The concentrations of cf-nDNA were calculated according to the standard curves, using known concentration of human genomic DNA (Roche) (final concentrations from 0.5 ng/mL to 5000 ng/mL r^2^ = 0.9707). The results were expressed as genome-equivalent (GE) per mL of plasma by using the conversion factor of 6.6 pg of DNA per cell. Genome equivalents were calculated as follows:

$${\rm{c}}={\rm{Q}}\times {{\rm{V}}}_{{\rm{DNA}}}/{{\rm{V}}}_{{\rm{PCR}}}\times 1/{{\rm{V}}}_{{\rm{EX}}}$$

For the calculation of the concentration (c) in genome equivalents (GE/mL) the DNA quantity (Q) obtained by qPCR was multiplied with one fraction consisting of the volume of eluted DNA (V_DNA_;) divided by the sample volume used for PCR (V_PCR_) and with another fraction consisting of the unit (1 ml) divided by the volume of extracted plasma (V_EX_).

The content of mtDNA was calculated using the delta Ct (ΔCt) of an average Ct of mtDNA and nDNA (ΔCt = Ct_nDNA_ − Ct_mtDNA_) in the same well as an exponent of 2 (2^ΔCt^). Relative quantities of cf-mtDNA could be estimated using an equation of GE (cf-nDNA) × fold-change cf-mtDNA and expressed also as GE per mL of plasma.

### Statistical analysis

All statistical analyses were performed using STATISTICA version 13.0 (StatSoft Inc. 2014). The Shapiro-Wilk test showed that our data were not normally distributed. Comparison between two groups was made using non-parametric Mann-Whitney test. Comparison between the three groups was made using Kruskal-Wallis test (non-parametric ANOVA). The receiver operating characteristic (ROC) curve was used for prediction of cut-off values of the markers studied. Numerical data were expressed as mean ± SD (median). p-values ≤ 0.05 were considered statistically significant.

### Ethical approval

All procedures performed in studies involving human participants were in accordance with the ethical standards of the institutional research committee (Bioethics Committee of the Medical University of Lodz for the research No. RNN/146/18/KE dated May 15th, 2018) and with the 1964 Helsinki Declaration and its later amendments or comparable ethical standards.

### Informed consent

Informed consent was obtained from all individual participants included in the study.

## Supplementary information

Supplementary

## Data Availability

The datasets generated during and/or analysed during the current study are available from the corresponding author on reasonable request.
