# Supplementary material for: Altered levels of circulating nuclear and mitochondrial DNA in patients with Papillary Thyroid Cancer
Source: Sci Rep. 2019 Oct 8;9:14438. doi: 10.1038/s41598-019-51000-7 (PMC6783406; doi:10.1038/s41598-019-51000-7)
Supplement: Supplementary file 1 — Supplementary [file 41598_2019_51000_MOESM1_ESM.pdf]

Supplementary material for

Altered levels of circulating nuclear and mitochondrial DNA in patients with Papillary Thyroid Cancer

Ewelina Perdas, Robert Stawski, Krzysztof Kaczka, Dariusz Nowak and Maria Zubrzycka

No.	Code	Gender	Age	T	N	M
1	1	M	33	T1	N1	M0
2	2	F	55	T1	N0	M0
3	9	M	50	T3	NX	M0
4	13	F	32	T3	N0	M0
5	14	F	70	T3	N0	M0
6	15	F	59	T1	N1	M0
7	16	F	42	T2	N0	M0
8	18	F	74	T1	N0	M0
9	20	F	76	T1	N1	M0
10	21	F	56	T1	N1	M0
11	24	F	44	T1	N1	M0
12	25	F	67	T1	N0	M0
13	27	F	63	T1	N0	M0
14	28	F	24	T1	N0	M0
15	29	F	49	T3	N1	M0
16	30	F	40	T1	N0	M0
17	31	F	30	T1	NX	M0
18	32	F	38	T3	N1	M0
19	33	F	42	T1	N0	M0
20	34	F	59	T1	NX	M0
21	35	F	67	T1	NX	M0
22	36	M	29	T2	NX	M0
23	37	F	38	T1	N0	M0
24	38	M	55	T3	N0	M0
25	39	F	29	T1	NX	M0
26	40	M	74	T4	NX	M0
27	41	F	20	T3	N1	M0
28	42	F	53	T1	N0	M0
29	43	F	41	T3	N1	M0
30	44	F	48	T2	N0	M0
31	45	F	46	T2	N0	M0
32	46	F	73	T1	NX	M0

Table 1S. Characteristics of PTC cases

No.	Code	Gender	Age
1	1C	F	67
2	2C	F	46
3	3C	F	47
4	4C	F	51
5	5C	F	66
6	6C	F	59
7	7C	F	47
8	8C	F	40
9	9C	F	65
10	10C	F	29
11	11C	F	56
12	12C	F	53
13	13C	F	54
14	14C	F	44
15	15C	M	46
16	16C	M	61
17	17C	M	41
18	18C	M	55
19	19C	M	40
20	20C	M	34
21	21C	M	47
22	22C	M	42
23	23C	M	37
24	24C	M	36
25	25C	F	30
26	26C	F	55
27	27C	M	52
28	28C	F	55
29	29C	F	45
30	30C	F	45

Table 2S. Characteristics of controls

GAPDH	5’-CCCCACACACATGCACTTACC-3’	forward primer
	5’-CCTAGTCCCAGGGCTTTGATT-3’	reverse primer
	5’-MGB-TAGGAAGGA CAGGCAAC–VIC-3’	probe

MT-ATP 8	5’-AATATTAAACACAAACTACCACCTAC C-3’	forward primer
	5’-TGGTTCTCAGGGTTTGTTATAA-3’	reverse primer
	5’-FAM-CCTCACCAAAGCCCATA-MG B-3’	probe

Table 3S. The primer and TaqMan probe sequence
